# Association between autoimmune diseases and mental disorders in children and adolescents

**DOI:** 10.1192/j.eurpsy.2023.1576

**Published:** 2023-07-19

**Authors:** Z. Elmaataoui, H. Belhadga, H. Kisra

**Affiliations:** Hospital AR-Razi of Sale, Sale, Morocco

## Abstract

**Introduction:**

Autoimmune diseases play a role in the etiology of a range of psychotic disorders which has attracted increasing interest in recent decades. This hypothesis is supported by genetic findings linking markers related to the immune system and mental disorders, and by clinical studies revealing increased levels of inflammatory markers in patients with mental disorders

**Objectives:**

The objective of our work is to discuss the involvement of autoimmune diseases in the development of mental disorders in children and adolescents.

**Methods:**

We conducted our study through the analysis of three clinical observations.

**Results:**

These were three patients followed in the child psychiatry department for a characterized depressive disorder, an ASD and an acute psychotic attack, and in whom an autoimmune disease was revealed either in the history or on paraclinical explorations.

**Conclusions:**

It appears legitimate to evoke and search for these autoimmune pathologies among the other organic etiologies, in front of any child or adolescent presenting acute and atypical psychiatric symptoms, tables of cognitive regression and resistances to the usual treatments. Biological immunological examinations, even invasive ones, should be repeated, if necessary.

**Disclosure of Interest:**

None Declared

